# Decoding Inflammation: Predicting Sepsis in the ICU

**DOI:** 10.7759/cureus.75256

**Published:** 2024-12-07

**Authors:** Balasubrahmanyam Chandrabhatla, Anitha A V, Lakshmi Sasidhar Puvvula, Palepu B Gopal

**Affiliations:** 1 Department of Critical Care Medicine, Citizens Specialty Hospital, Hyderabad, IND

**Keywords:** biomarkers in sepsis, early sepsis, inflammatory biomarker, inflammatory biomarkers for the development of sepsis in icu, mean monocyte volume (mmv), monocyte distribution width (mdw), resource limited setting, sepsis prediction

## Abstract

Background: Sepsis is a life-threatening condition arising from a dysregulated host response to infection leading to organ dysfunction. Traditional clinical signs are often unreliable for detecting sepsis, necessitating the exploration of more accurate biomarkers. Furthermore, currently, recommended screening scores perform poorly, necessitating more effective biomarkers to identify sepsis. Therefore, in this study, we evaluated the predictive capabilities of six inflammatory biomarkers - C-reactive protein (CRP), red cell distribution width (RDW), neutrophil-lymphocyte ratio (NLR), monocyte distribution width (MDW), mean neutrophil volume (MNV), and mean monocyte volume (MMV) - measured from samples taken for complete blood count (CBC) for the development of sepsis in ICU patients.

Methods: We conducted a prospective observational study involving ICU patients at a tertiary-care hospital in Hyderabad, India, over a one-year period to primarily assess the predictability of these six biomarkers for sepsis. As a secondary outcome, we also analyzed the predictiveness of the biomarkers with respect to mortality, the need for vasopressors, invasive mechanical ventilation or renal replacement therapy (RRT), the presence of organ failure, and hospital length of stay. Blood samples were collected for CRP and CBC on the first day of admission, from which RDW, NLR, MDW, MNV, and MMV were measured. Demographic data, including Acute Physiology and Chronic Health Evaluation II (APACHE II) and Sequential Organ Failure Assessment (SOFA) scores, clinical progression (recovery or mortality), requirement for vasopressors, invasive mechanical ventilation, RRT, presence of organ failure, and overall length of hospital stay, were documented.

Results: We analyzed data from 84 patients after one patient withdrew consent. The study sample had a mean age of 65.83 years, with 60 (71.4%) patients >60 years of age and a male predominance (n = 50; 59.5%). About 57 (67.85%) patients had three or more comorbidities. About 71 (84.5%) patients met the sepsis-3 criteria. Mean APACHE II and SOFA scores were 18.73 and 5.36, respectively. Primary outcome analysis showed that CRP and MDW were the most sensitive, with sensitivities of 81.75% and 81.7%, respectively, whereas MDW, MNV, and MMV had the highest specificity at 100% each. Correlation analysis revealed that MDW had the best area under the curve (AUC) of 0.932 in predicting sepsis. Multivariate logistic regression identified both MDW and MMV to have a significant positive correlation in the prediction of sepsis. The overall mortality rate was 9.5%. About 82 (97.6%) patients had organ failure, 35 (41.7%) required vasopressors, 20 (23.8%) required invasive mechanical ventilation, 16 (19%) required RRT, and 59 (70.2%) had a hospital stay exceeding five days, with an average length of hospital stay of eight days. No biomarkers showed strong AUC or specificity compared to the SOFA score (0.710, 94.74% specificity in predicting mortality). However, among the six biomarkers, MDW was the most specific (86.84%). A CRP of >65 mg/L was the best indicator for prolonged hospital stay, vasopressor use, and RRT. An MMV of >179 and MDW of >21.86 U were the most sensitive markers for vasopressor requirements.

Conclusion: Our findings suggest that easily accessible biomarkers derived from routine CBC tests, particularly MDW and MMV, may serve as valuable tools for early sepsis diagnosis in resource-limited settings.

## Introduction

Sepsis is defined as a life-threatening organ dysfunction caused by dysregulated host response to infection [1]. Organ dysfunction is defined by a sequential (sepsis-related) organ failure assessment (SOFA) score of more than 2 [2]. Sepsis and septic shock are leading causes of mortality in the ICU. Lack of sepsis recognition is one of the common causes of its misdiagnosis and contributes to adverse outcomes due to delays in definitive antimicrobial treatments. Every hour that treatment is delayed is associated with a 7%-10% increase in sepsis-related mortality [3]. Furthermore, prognostic scores for sepsis have been found to be unsatisfactory, highlighting an urgent need for more effective tools and biomarkers to identify sepsis [4].

There is a large unmet clinical need for early detection of patients with sepsis. C-reactive protein (CRP) has been shown to be a more sensitive and specific biomarker than white blood cell (WBC) count [5]. Monocyte distribution width (MDW) is a parameter derived from a standard complete blood count (CBC) that reflects the size distribution of circulating monocytes, which play a crucial role as the first line of defense against infections. The measurement of the mean monocyte volume (MMV) and mean neutrophil volume (MNV) are emerging as potential indicators that aid clinicians in diagnosing sepsis. Additionally, higher MNV, MMV values, and MDW values have been observed in acute bacterial infections, particularly in bloodstream infections caused by Gram-negative bacteria. The neutrophil-lymphocyte ratio (NLR) can be calculated using either the absolute counts of neutrophils and lymphocytes or their relative proportions. Its significance lies in the observation that physiological stress typically leads to an elevation in neutrophil counts and a reduction in lymphocyte counts. In cases of sepsis, there is an increase in lymphocyte apoptosis, resulting in a higher NLR. During septic shock, there is a marked decrease in lymphocyte numbers, which causes a significant rise in the NLR. It has been suggested that inflammation and oxidative stress can decrease red blood cell (RBC) survival and hinder their maturation. This disruption may lead to the release of larger, more immature RBCs into the bloodstream, which in turn contributes to an elevated red cell distribution width (RDW). Procalcitonin is used to differentiate bacterial infections from non-infectious causes and viral infections [6] and shows higher predictive sensitivity than CRP [7]. Like procalcitonin, many other biomarkers of sepsis, such as sTREM-1 [8], presepsin [9], proadrenomedullin [10], and proenkephalin [11], are not widely available, and testing is expensive.

Many of our patients are of low or middle socioeconomic status, making the above-mentioned biomarkers prohibitively expensive or unavailable for testing. Traditionally, leukocytosis is considered indicative of sepsis, but the condition is non-specific. CBC testing is easily available and inexpensive. Newer automated blood count analyzers (e.g., Beckman Coulter-LH series) use volume, conductivity, and scatter (VCS) technology to provide differential white-cell analysis. Furthermore, parameters such as red cell distribution width (RDW), MDW, mean monocyte volume (MMV), NLR, and MNV can be determined, which have been of recent interest for early diagnosis of sepsis, as neutrophils and monocytes represent the first line of defense against invading pathogens [12]. Therefore, in this prospective observational study, we aimed to analyze these parameters (available from CBC and CRP) for the prediction of sepsis and outcomes in the ICU.

## Materials and methods

Study design

We conducted a prospective observational study in the medical ICU at Citizens Specialty Hospital in Hyderabad, India, over a duration of one year from May 2021 to April 2022. Blood samples for CBC were collected as part of routine practice, sent to the central laboratory, and tested using a Beckman Coulter LH 750 hematology analyzer (Beckman Coulter, Inc., USA). Standard care protocols were followed for all patients throughout the study period. The study was approved by the Institutional Ethics Committee of Citizens Specialty Hospital (approval number: ECR/1103/Inst/TG/2018/RR-21).

Study population

The study population comprised patients admitted to the ICU regardless of diagnosis. The study included all patients aged 18 years and older, with the sole exclusion criterion being age under 18 years.

Data collection

Data were collected using a structured data sheet to gather pertinent patient information. All necessary investigations were sent, including CBC, CRP, and cultures when necessary. Within the first 24 hours of admission, the following biomarkers were measured from venous blood samples: CRP, RDW, NLR, MDW, MMV, and MNV. Acute Physiology and Chronic Health Evaluation II (APACHE II) and SOFA scores were calculated within the first 24 hours of admission using an online scoring calculator. Additionally, clinical progression was closely monitored throughout each patient’s hospital stay, documenting outcomes such as recovery or death, presence of one or more organ failures, length of hospital stay, requirements for ventilation, vasopressor use, renal replacement therapy (RRT), and total length of hospital stay.

Data analysis

IBM SPSS Statistics for Windows, Version 20 (Released 2011; IBM Corp., Armonk, New York, United States) was used to analyze the collected data. Means and standard deviations for various biomarkers were calculated. Correlations between independent groups were determined using Pearson correlation or Fisher’s exact test, whereas multivariate logistic regression was conducted to assess all biomarkers in relation to sepsis prediction. The chi-square test was used to compare categorical variables. To evaluate the discrimination performance of all biomarkers concerning sepsis and mortality predictions, a receiver operating characteristic (ROC) curve was generated, and sensitivity and specificity values were calculated. A p-value of less than 0.05 was considered statistically significant.

Sample size calculation

Based on a previous study by Sakr et al. [13], which estimated sepsis occurrence in ICU patients at 29.5%, a sample size of 79 was calculated:

\[ n = \frac{P(1-P)(Z_\alpha)^2}{ME^2} \]

where n is the sample size, P is sepsis prevalence (29.5%), Z_α_ is at a 5% level of significance in a standard normal distribution (1.96), and ME is marginal error (10%).

\[ n = 29 \times 71 \times (1.96)^2 / (10)^2 \]

\[n = 79.09\]

Therefore, an additional 10% was included for potential losses, resulting in a final sample size of 85 patients. Of the 85 patients who were enrolled in the study, one patient withdrew consent. Therefore, data from 84 patients were analyzed.

## Results

The study population had a mean age (±standard deviation) of 65.83 (±12.62) years, with the majority of patients (n = 60, 71.4%) aged >60 years. Of the 84 patients who were analyzed, 50 (59.5%) were male, and 34 (40.5%) were female; 57 (67.85%) patients had three or more comorbidities. Using the sepsis-3 definition criteria (clinical suspicion + acute increase in SOFA by ≥2), 71 (84.5%) patients were found to have sepsis. Mean APACHE II and SOFA scores were 18.73 and 5.36, respectively (Table 1).

**Table 1:** Demographic information of study population SD: standard deviation; %: percentage; APACHE II: Acute Physiology and Chronic Health Evaluation II; SOFA: Sequential Organ Failure Assessment *Sepsis defined according to the sepsis-3 definition (clinical suspicion + acute increase in SOFA by ≥2 points)

Demographic variable	Value
Age (years), mean ± SD	65.83 ± 12.62
Male sex, n (%)	50 (59.5%)
≥3 comorbid conditions, n (%)	57 (67.85%)
APACHE II score, mean ± SD	18.73 ± 7.99
SOFA score, mean ± SD	5.38 ± 2.62
Sepsis, n (%)*	71 (84.5%)

The mean values of the biomarkers are presented in Table 2.

**Table 2:** Mean values of biomarkers in study population SD: standard deviation; mg/L: milligrams per liter; CV: coefficient of variation; CRP: C-reactive protein; RDW: red cell distribution width; NLR: neutrophil lymphocyte ratio; MDW: monocyte distribution width; MNV: mean neutrophil volume; MMV: mean monocyte volume MDW is expressed as U-Units ^$^NLR is a ratio that has no units, MNV and MMV are CV % have no units; *No universally accepted cutoff values, range taken from prior studies cut-off values

Biomarker	Mean ± SD	Normal values
CRP (mg/L)	159.41 ± 121.77	0-6
RDW (CV)	15.77 ± 2.18	11.6-14.6
NLR^$^	16.67 ± 12.23	1-3
MDW (U)	25.70 ± 5.53	17-20*
MNV^$^	158.64 ± 13.06	138-146.5*
MMV^$^	183.85 ± 15.94	158-172*

Primary outcome

**Figure 1:**
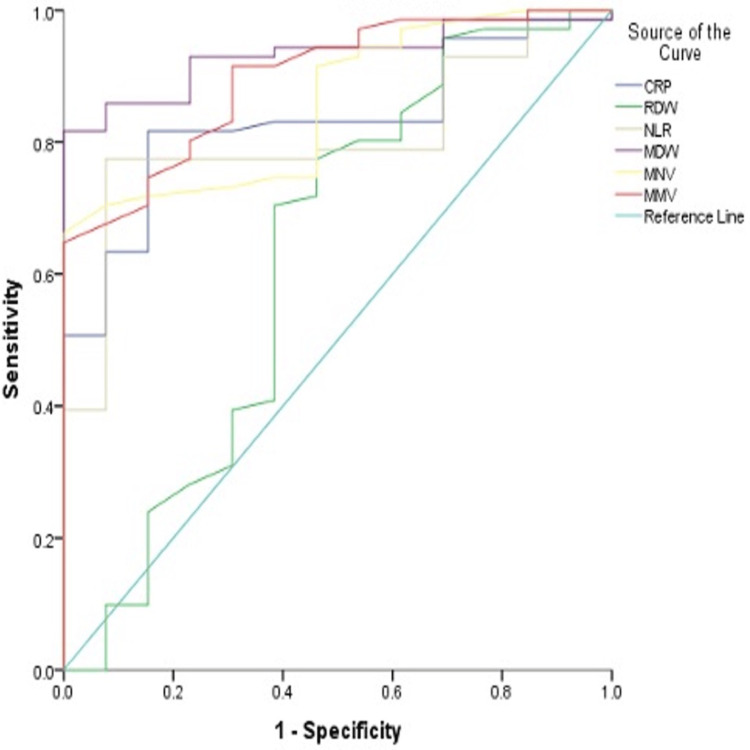
Receiver operating characteristic curves for sepsis prediction of all biomarkers CRP: C-reactive protein; RDW: red cell distribution width; NLR: neutrophil-lymphocyte ratio; MDW: monocyte distribution width; MNV: mean neutrophil volume; MMV: mean monocyte volume

In our study, the most sensitive markers for sepsis prediction were CRP (81.75%) and MDW (81.7%), whereas the most specific markers were MDW, MNV, and MMV, with 100% specificity for each. Overall, MDW had good sensitivity, specificity, and the best AUC (0.932) when compared to the rest of the biomarkers (Table 3 and Figure 1).

**Table 3:** Primary outcome: predictability of sepsis by biomarkers AUC: area under the curve; CI-LB: confidence interval-lower boundary; CI-UB: confidence interval-upper boundary; CRP: C-reactive protein; RDW: red cell distribution width; NLR: neutrophil-lymphocyte ratio; MDW: monocyte distribution width; MNV: mean neutrophil volume; MMV: mean monocyte volume

Biomarker	AUC	CI-LB	CI-UB	Cut-off	Sensitivity	Specificity
CRP	0.832	0.735	0.928	65.95	81.75%	84.6%
RDW	0.626	0.430	0.832	14.65	70.4%	61.5%
NLR	0.807	0.701	0.913	7.95	77%	92%
MDW	0.932	0.878	0.985	21.86	81.7%	100%
MNV	0.858	0.769	0.947	155.2	66.2%	100%
MMV	0.897	0.819	0.974	179	64.8%	100%

Our correlation analysis showed that except for RDW, all biomarkers showed a significant positive correlation in the prediction of sepsis (Table 4).

**Table 4:** Correlation analysis of sepsis prediction for all biomarkers CRP: C-reactive protein; RDW: red cell distribution width; NLR: neutrophil-lymphocyte ratio; MDW: monocyte distribution width; MNV: mean neutrophil volume; MMV: mean monocyte volume

	CRP	RDW	NLR	MDW	MNV	MMV
Pearson correlation	<0.001	0.026	<0.001	<0.001	<0.001	<0.001
Fischer’s exact t-test	<0.001	0.053	<0.001	<0.001	<0.001	<0.001

According to subsequent multivariate logistic regression analysis, only MDW and MMV were statistically significant in the prediction of sepsis (Table 5).

**Table 5:** Multivariate regression analysis via two-stage least squares method df: degrees of freedom; CRP: C-reactive protein; RDW: red cell distribution width; NLR: neutrophil-lymphocyte ratio; MDW: monocyte distribution width; MNV: mean neutrophil volume; MMV: mean monocyte volume

Biomarker	Score	df	p-value
CRP	0.240	1	0.624
RDW	0.102	1	0.749
NLR	1.393	1	0.238
MDW	0.571	1	0.028
MNV	0.000	1	0.990
MMV	0.121	1	0.046
Overall	1.830	4	0.767

Secondary outcome

The study population had an overall mortality of 9.5%, representing eight individuals. About 82 (97.6%) patients had one or more organ failures, 35 (41.7%) required a vasopressor, 20 (23.8%) required invasive mechanical ventilation, and 16 (19%) required RRT. Mean hospital stay was approximately eight days, and 59 (70.2%) patients required a hospital length of stay of more than five days (Table 6).

**Table 6:** Incidence of secondary outcomes in study population SD: standard deviation, %: percentage; IMV: invasive mechanical ventilation; RRT: renal replacement therapy; LOS: length of stay

Secondary outcome	Incidence, n (%)
Mortality	8 (9.5%)
Presence of ≥1 organ failure	82 (97.6%)
Vasopressor requirement	35 (41.7%)
Need for IMV	20 (23.8%)
Need for RRT	16 (19%)
Hospital LOS (days), mean ± SD	8 ± 4.8
Hospital LOS >5 days	59 (70.2%)

**Figure 2:**
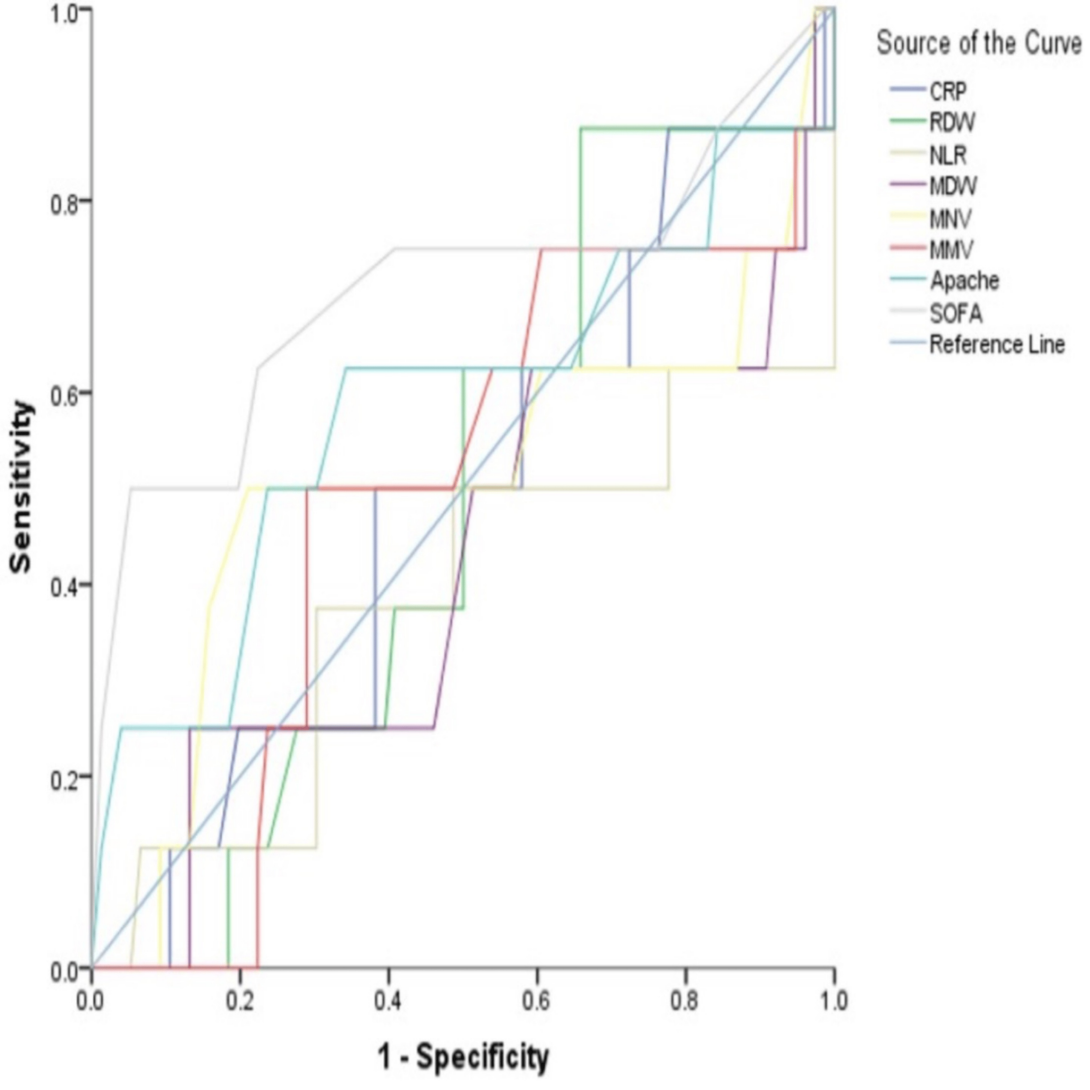
Receiver operating characteristic curves for sepsis prediction of all biomarkers CRP: C-reactive protein; RDW: red cell distribution width; NLR: neutrophil-lymphocyte ratio; MDW: monocyte distribution width; MNV: mean neutrophil volume; MMV: mean monocyte volume; SOFA: Sequential Organ Failure Assessment; APACHE: Acute Physiology and Chronic Health Evaluation

After analyzing the secondary outcomes in our study, we found that the SOFA score had the best AUC (0.710) and specificity (94.74%) for mortality prediction when compared to all other biomarkers (Figure 2).

MDW was found to be the most specific biomarker (86.84%) (Table 7).

**Table 7:** Secondary outcome: comparison of mortality prediction by biomarkers and APACHE II and SOFA scores CRP: C-reactive protein; RDW: red cell distribution width; NLR: neutrophil-lymphocyte ratio; MDW: monocyte distribution width; MNV: mean neutrophil volume; MMV: mean monocyte volume; APACHE II: Acute Physiology and Chronic Health Evaluation II; SOFA: Sequential Organ Failure Assessment

Biomarker	AUC	Sensitivity	Specificity
				CRP	0.486	50.00%	61.80%
RDW	0.480	87.50%	61.84%
NLR	0.384	37.50%	69.74%
MDW	0.417	25.00%	86.84%
MNV	0.508	50.00%	78.95%
MMV	0.489	50.00%	71.05%
APACHE II	0.589	62.50%	65.79%
SOFA	0.710	50.00%	94.74%

Based on the cutoff values derived from ROC curves for the prediction of other secondary outcomes, the majority of biomarkers were able to predict vasopressor requirement, but the most sensitive markers were MMV (>179) and MDW (>21.86 U). MMV (>179) was the most sensitive predictor for the need for RRT, followed by CRP (>65.95 mg/L), with the latter also being the best predictor for hospital length of stay exceeding five days. RDW (>14 fL) and NLR (>7.95) were able to predict the occurrence of one organ failure (Table 8).

**Table 8:** Remaining secondary outcome predictions by biomarkers CRP: C-reactive protein; RDW: red cell distribution width; NLR: neutrophil-lymphocyte ratio; MDW: monocyte distribution width; MNV: mean neutrophil volume; MMV: mean monocyte volume; IMV: invasive mechanical ventilation; RRT: renal replacement therapy; CV: coefficient of variation ^$^NLR is a unitless ratio. MNV and MMV have no units

Biomarker	Hospital stay	Organ failure	Vaso pressors	IMV	RRT
CRP > 65.95 (mg/L)	0.010	0.497	0.014	0.685	0.028
RDW > 14 (CV)	0.492	0.049	0.332	0.035	0.008
NLR > 7.95^$^	0.063	0.043	0.008	0.365	0.169
MDW > 21.86 (U)	0.892	0.550	0.005	0.316	0.076
MNV > 155.2^$^	0.635	0.864	0.016	0.676	0.088
MMV > 179.0^$^	0.268	0.891	0.002	0.590	0.018

## Discussion

A wide array of biomarkers has been proposed for sepsis, far surpassing those identified for other conditions. For example, research on myocardial infarction identified 14 suitable biomarkers [14], whereas only eight were found for Alzheimer’s disease [15]. This substantial difference is likely due to the intricate pathophysiology associated with sepsis, which involves numerous inflammatory mediators and other complex mechanisms [16]. Given the serious implications of sepsis, early detection in the ICU is crucial. This study aimed to evaluate the predictive value of inflammatory markers for the development of sepsis in ICU patients.

In our study, we examined 84 patients, with the study population being predominantly male (59.5%). We found that 71 (84.5%) patients were clinically suspected of having sepsis, among whom 57 (80.2%) had positive bacteriological cultures. We assessed six biomarkers for their effectiveness in predicting sepsis in ICU admissions: CRP, RDW, NLR, MDW, MNV, and MMV. The most sensitive biomarkers for predicting sepsis were CRP (81.75%) and MDW (81.7%). The most specific markers were MDW, MNV, and MMV, at 100% each.

Our findings indicated that CRP, with a cutoff value of 65.95, showed a sensitivity of 81.75% and a specificity of 84.6%, with an AUC of 0.832, indicating a significant positive correlation in predicting sepsis. This result aligns with previous research by Yang et al. [17]. Conversely, RDW did not demonstrate a significant correlation at a cutoff of 14.65, which contrasts with findings by Zhang et al. [18], who reported a significant correlation with a cutoff of 13.45. NLR showed a significant positive correlation in predicting sepsis with a cutoff of 7.95, although a higher cutoff (>12.1) was observed in a study by Lorente et al. [19]. MDW at a cutoff of 21.86 exhibited a sensitivity of 81% and a specificity of 100% (AUC = 0.932), indicating a significant predictive capacity for sepsis. Similar results were reported by Crouser et al. [20] and Jo et al. [21] with cutoffs of 21.85 and 21.85, respectively. MNV, with a cutoff value of 155.5, demonstrated a sensitivity of 66.2% and a specificity of 100% (AUC = 0.858), corroborating findings by Suresh et al. [22], who noted an increased MNV for both systemic and localized infections compared to controls, with a cutoff of 158.3 ± 13.7. MMV, at a cutoff of 179, showed a sensitivity of 64% and specificity of 100% (AUC = 0.897); similar outcomes were noted in studies by Arora et al. [23] and Lee and Kim [24], where MMV cutoffs of 168.3 and 181.5 resulted in sensitivities of 80.6% and 77.8% and specificities of 77.5% and 74.6%, respectively.

Based on our analysis, CRP and MDW exhibited good sensitivity and specificity compared to other biomarkers, with MDW achieving the highest AUC of 0.932. Five out of the six studied biomarkers indicated a significant positive correlation in predicting sepsis. To further confirm these correlations, we conducted a multivariate regression analysis using two-stage least square regression, which revealed that MDW and MMV had statistically significant positive correlations with sepsis prediction, with p-values of 0.028 and 0.046, respectively.

Regarding secondary outcomes, although our study was not designed to assess mortality differences, we aimed to determine whether any biomarkers showed a comparable or superior correlation to APACHE II and SOFA scores in predicting mortality. We found that no biomarkers surpassed the SOFA score, which had an AUC of 0.710 and a specificity of 94.74%. However, MDW (86.84%) was the most specific among the studied biomarkers. CRP, with a cutoff value exceeding 65.95 mg/L, has been identified as the most reliable predictor for prolonged hospital stays, the need for vasopressors, and the requirement for RRT, consistent with the findings of Han et al. [25]. Although many biomarkers can predict the need for vasopressors, the most sensitive markers identified were MMV (>179) and MDW (>21.86 U) [26]. Specifically, MMV at a cutoff of >179 was determined to be the strongest indicator for requiring RRT. Additionally, CRP (>65.95 mg/L) was the primary predictor for hospital stays lasting more than five days. In addition, RDW (>14 fL) and NLR (>7.95) were significant predictors of experiencing one or more organ failures. Zhu et al. [27] demonstrated that higher levels of RDW and NLR correlated with an increased incidence of multiple organ failure in critically ill patients.

Wu et al. [28] reported that a combination of MDW and other inflammatory indicators (e.g., procalcitonin, CRP, interleukin, and leukocyte-related indicators) has great potential for early diagnosis and prediction of sepsis prognosis, making MDW a promising new biomarker for sepsis diagnosis and prognosis.

This study explored the predictive capacities of inflammatory markers for sepsis, highlighting the recent emphasis on MDW, MNV, and MMV. Our results indicated that MDW and MMV were the most effective markers for predicting sepsis. Given that these markers can be derived from a CBC, which is an inexpensive and routine test, they represent valuable tools in resource-limited settings where they are readily accessible and do not impose additional costs on patients; their availability for rapid diagnosis of sepsis allows for timely initiation of antibiotic therapy.

Study limitations

Our results should be interpreted within the context of certain limitations. This study did not demonstrate statistically significant differences in mortality prediction and was not sufficiently powered for this outcome. A larger sample size could potentially enhance the ability to predict mortality and other secondary objectives.

## Conclusions

Our study reinforces the critical role of inflammatory biomarkers in the early detection and prediction of sepsis in ICU patients, identifying CRP and MDW as the most sensitive and specific indicators, with MDW demonstrating a high predictive capacity. These biomarkers, when used alongside established clinical scoring systems like APACHE II and SOFA, can significantly improve the early identification of sepsis, facilitating timely interventions, which are crucial for improving patient outcomes. The ability to derive MDW and MMV from routine CBCs offers substantial benefits for healthcare systems, particularly in resource-limited settings, enabling rapid and cost-effective diagnostics.
